# Age‐Dependent Efficacy of Electroconvulsive Therapy in Depression: A Longitudinal Study of Symptom Trajectories and Treatment Predictions

**DOI:** 10.1002/brb3.70487

**Published:** 2025-04-21

**Authors:** Wanling Huang, Yang Ji, Nanxue Duan, Hao Zheng, Rui Qian, Kai Wang, Jianhong Li, Yanghua Tian

**Affiliations:** ^1^ Department of Neurology The First Affiliated Hospital of Anhui Medical University Hefei China; ^2^ Anhui Province Key Laboratory of Cognition and Neuropsychiatric Disorders Hefei China; ^3^ The College of Mental Health and Psychological Sciences Anhui Medical University Hefei China; ^4^ Collaborative Innovation Center of Neuropsychiatric Disorders and Mental Health Hefei China; ^5^ Department of Mental Health First Hospital of Shanxi Medical University Taiyuan China; ^6^ Department of Psychology and Sleep Medicine The Second Affiliated Hospital of Anhui Medical University Hefei China

**Keywords:** age differences, depression scores, electroconvulsive therapy, personalized treatment, symptom dimensions, treatment prediction

## Abstract

**Background:**

The variability in electroconvulsive therapy (ECT) efficacy for depression has received significant attention, particularly regarding the influence of age on treatment outcomes. However, studies examining the trajectory of ECT efficacy across different age groups remain limited.

**Methods:**

This longitudinal study analyzed 1056 intensive longitudinal data measurements from 132 hospitalized patients diagnosed with major depressive episodes, categorized into a young group (<30 years, *n* = 69) and an older group (≥30 years, *n* = 63). Depression severity was assessed using the Hamilton Depression Rating Scale‐17 (HAMD‐17) at baseline and within 24 h after each ECT session, with data collected for up to eight treatments. Statistical analyses included linear mixed‐effects model (LMM), survival analysis, and linear regression to evaluate the impact of age on ECT efficacy, the predictive ability of depression scores, and differences in symptom dimension improvements (core depressive symptoms, anxiety, insomnia, and somatic symptoms).

**Results:**

LMM analysis revealed that each ECT session significantly reduced total HAMD‐17 scores (*β* = −2.166, *p* < 0.001), with greater improvement observed in the older group (*β* = 0.463, *p* < 0.001). Symptom dimension analysis showed significant reductions in core depressive symptoms, anxiety, insomnia, and somatic symptoms, with greater improvement in anxiety and somatic symptoms in older patients. Kaplan–Meier analysis showed that the older group achieved remission with fewer sessions than the younger group (median: 5 vs. 7, *χ* ^2^ = 4.100, *p* = 0.042). However, Cox regression identified baseline HAMD‐17 scores (hazards ratio [HR] = 0.945, 95% confidence interval [CI]: 0.914–0.977, *p* = 0.001) and the use of serotonin–norepinephrine reuptake inhibitors (HR = 1.52, 95% CI: 1.075–2.151, *p* = 0.018) as significant predictors of remission, thereby eliminating the initial group difference. In the older group, both baseline and first post‐ECT HAMD‐17 scores predicted the number of sessions required (*R* ^2^ = 0.125, *p* = 0.016; *R* ^2^ = 0.134, *p* = 0.017), whereas in the younger group, only first post‐ECT scores were predictive (*R* ^2^ = 0.282, *p* = 0.001).

**Conclusion:**

Age significantly influences ECT efficacy and the prediction of treatment requirements, with older patients exhibiting better responsiveness. Initial depression scores effectively predict the number of treatments required, supporting the need for age‐specific, personalized ECT treatment strategies.

## Graphical abstract

Age significantly influences ECT remission speed and the prediction of treatment requirements in patients with depression, with older patients (age ≥ 30) showing better responses.

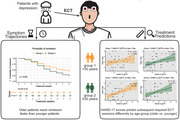

## 1 Introduction

Depression is a widespread psychological disorder that poses significant challenges to public health due to its high incidence, high recurrence rate, and substantial disability burden (Smith 2014). Despite the availability of various pharmacological and psychotherapeutic interventions, a considerable proportion of patients with major depressive disorder exhibit treatment‐resistant depression, necessitating alternative therapeutic approaches (McIntyre et al. 2023). Electroconvulsive therapy (ECT) has long been recognized as one of the most effective treatments for severe and refractory depression, demonstrating rapid and substantial improvements in depressive symptoms (Kellner et al. 2012). ECT is particularly indicated for patients experiencing severe depressive episodes, psychotic features, or an imminent suicide risk, providing a crucial intervention when other treatments are ineffective (Anand et al. 2023; Fink et al. 2014; Yrondi et al. 2024).

However, the efficacy of ECT is not uniform across patient populations, with growing evidence indicating significant heterogeneity in treatment responses (Haq et al. 2015; Wade et al. 2021). The factors influencing its effectiveness are complex and multifaceted (Dai et al. 2024; Lihua et al. 2014; Minelli et al. 2016). Among these factors, age has emerged as a significant variable of interest. However, the literature on the effect of age on ECT outcomes is inconsistent. Some studies suggest that ECT is more effective in older patients than in younger ones (O'Connor et al. 2001), potentially due to the higher prevalence of psychomotor retardation in older individuals (Heijnen et al. 2019). Conversely, other studies have indicated that although older age is associated with better ECT outcomes, this relationship is primarily mediated by core depressive symptoms, such as psychomotor agitation, retardation, and/or psychotic features (van Diermen et al. 2020).

Furthermore, structural and functional changes in the brain associated with aging may affect the efficacy of ECT. Degenerative changes, including medial temporal lobe atrophy and hippocampal volume reduction, may reduce the responsiveness of older patients to ECT (Oudega et al. 2011). However, other studies have reported that older age may predict better outcomes (van Diermen et al. 2018). A retrospective study in the Netherlands that analyzed a decade of ECT treatment data found that older patients with age‐related brain lesions responded well to ECT (Sienaert et al. 2017). Conversely, other studies have found no significant relationship between age and ECT efficacy (Antosik‐Wójci and Święcicki 2016; Luccarelli et al. 2022). Although the relationship between age and ECT outcomes has been extensively studied, the results remain inconsistent, and the mechanisms by which age influences ECT efficacy remain unclear.

Significant heterogeneity exists among patients experiencing depressive episodes, which may lead to variations in the efficacy of ECT (Heikman et al. 2002). The symptom dimensions outlined by the Hamilton Depression Rating Scale (HAMD) can help identify this heterogeneity (Shafer 2006). Studies have shown that analyzing different HAMD symptom dimensions can reveal that changes in these dimensions following ECT intervention may be associated with alterations in distinct brain regions (Wade et al. 2021). Age may exert different effects on various brain regions, leading to differences in ECT efficacy. Therefore, it is necessary to further investigate the trajectory of changes in these symptom dimensions with ECT intervention across different age groups.

Accurate prediction of a patient response to ECT is crucial for optimizing treatment strategies owing to the side effects associated with ECT and its highly individualized therapeutic outcomes (Van der et al. 2023). Numerous clinical indicators, such as the presence of psychosis, high suicide risk, treatment resistance, and illness chronicity, have been reported to predict the efficacy of ECT (Dombrovski et al. 2005; Pinna et al. 2018). However, these indicators have shown inconsistent results across studies (Tsuchiyama et al. 2005), possibly due to patient heterogeneity. Variability among patients of different ages may complicate the prediction of ECT efficacy based on these indicators. However, it remains unclear whether age further complicates this predictive process. Currently, there is a lack of research on whether age affects the ability of clinical indicators (e.g., depression scores) to predict the need for subsequent treatment.

On the basis of these considerations, this study proposes the following hypotheses:

**Hypothesis 1**. *Older patients will exhibit a greater reduction in 17‐item Hamilton Depression Rating Scale [HAMD‐17] scores and require fewer ECT sessions to achieve remission than younger patients*.
**Hypothesis 2**. *Significant differences exist in the improvement of symptom dimensions (core depressive symptoms, anxiety, insomnia, and somatic symptoms) between older and younger patients undergoing ECT*.
**Hypothesis 3**. *Baseline and post‐first ECT HAMD‐17 scores significantly predict the number of ECT sessions required for remission, with variations between age groups*.

To test these hypotheses, this longitudinal study aimed to evaluate the impact of age on ECT efficacy by analyzing changes in HAMD‐17 scores after each ECT session and determining the number of sessions needed for remission in both young and older patient groups. Additionally, it examined differences in symptom dimension improvements between these age groups and assessed the predictive ability of depression scores (including baseline and post‐first ECT) in determining the required number of ECT treatments. This study aimed to develop age‐specific, personalized ECT treatment protocols, thereby enhancing therapeutic outcomes and improving the quality of life of individuals with major depressive episodes.

## 2 Methods and Materials

### 2.1 Study Design

This longitudinal study recruited patients with depression who underwent ECT at the Anhui Mental Health Center (AMHC) between January 2023 and June 2024. The severity of depression was assessed using HAMD‐17 (Park et al. 2014). On the basis of previous research, the total depression score was divided into four dimensions (Shafer 2006): Dimension 1 (core depressive symptoms, including items 1, 2, 3, 7, and 8); Dimension 2 (anxiety, including items 9, 10, 11, 15, and 17); Dimension 3 (insomnia, including items 4, 5, and 6); and Dimension 4 (somatic symptoms, including items 12, 13, 14, and 16). All patients were assessed using the HAMD‐17 at baseline (the day before the first ECT session) and within 24 h of each subsequent ECT session, up to the 8th session. For those who underwent more than eight ECT sessions, only the HAMD‐17 scores from the first eight sessions were recorded to ensure consistency in the evaluations. The patients were categorized into younger and older groups on the basis of their median age (the younger 50% and older 50%) (Bustillo et al. 2019). We documented and analyzed the baseline characteristics of both groups and examined changes in depressive symptoms throughout the course of ECT treatment. Remission was defined as an HAMD‐17 score of less than 7.

### 2.2 Participants

Participants were recruited from the inpatient department of the AMHC. The inclusion criteria included experiencing a major depressive episode as defined by Diagnostic and Statistical Manual of Mental Disorders‐5 (DSM‐5) criteria (diagnosis confirmed by two psychiatrists, encompassing both unipolar and bipolar depression), indication for ECT treatment, age between 16 and 65 years, the ability to complete the ECT course, and provision of written informed consent. The exclusion criteria were the presence of comorbid psychiatric disorders (such as substance abuse or schizoaffective disorder), current severe physical illness, a history of neurological disorders (such as traumatic brain injury or dementia), low educational attainment, or having undergone ECT within the past 6 months. During the study period, from January 2023 to June 2024, a total of 180 patients met the initial inclusion criteria. However, 28 patients were excluded for not meeting all criteria, and 20 withdrew from the study. Ultimately, 132 patients completed all 8 assessments. All participants provided written informed consent.

### 2.3 ECT Procedure

Modified bifrontal ECT was administered in accordance with the “Chinese Expert Consensus on Modified Electroconvulsive Therapy (2019 Edition)” guidelines, using the Thymatron IV Integrated ECT System (Somatics, Lake Bluff, IL, USA). Anesthesia was induced with propofol (1.4 mg/kg), and muscle relaxation was achieved using succinylcholine (0.5 mg/kg). Atropine (0.5 mg) was administered to suppress glandular secretions. Physiological monitoring included pulse oximetry and electrocardiography. The initial stimulus dose was determined using the half‐age method (Abrams 2002b), with stimulation parameters, including bifrontal electrode placement, a constant current of 0.9 A, and a pulse width of 0.5 ms. Both motor responses and electroencephalographic (EEG) seizure durations were closely monitored. To ensure a seizure duration of at least 25 s (Abrams 2002a), adjustments to the stimulus dose were made as necessary. The first three ECT sessions were administered consecutively, with subsequent sessions scheduled every other day, excluding weekends (Wang et al. 2020). If a seizure lasted less than 25 s, we followed the Chinese Expert Consensus on Modified ECT (2019 Edition), which recommends increasing the stimulation energy by 5% (approximately 25 mC in the Thymatron system) until a therapeutically satisfactory seizure was achieved. Throughout the ECT treatment period, the medication regimens of all patients remained unchanged.

### 2.4 Statistical Analysis

This study evaluated the trajectory of depression score changes in two groups of patients with depressive episodes after undergoing eight ECT sessions. The following statistical analyses were conducted.

#### 2.4.1 Descriptive Statistics and Baseline Comparisons

Continuous variables (e.g., baseline depression scores) were compared using independent sample *t*‐tests. Categorical variables (e.g., gender, medical history) were compared using chi‐square (*χ* ^2^) tests. Baseline indicators with significant differences were considered potential confounding factors in subsequent models.

#### 2.4.2 Linear Mixed‐Effects Model (LMM) Analysis

The HAMD‐17 score was used as the primary outcome measure to evaluate the effects of varying numbers of ECT sessions on depression symptom remission and associated factors in the two groups. An LMM (Whitlock et al. 2017) was employed to examine the effect of treatment sessions on HAMD‐17 scores and the interaction between groups. To capture the dynamic changes in scores over the treatment course, treatment sessions were included as fixed effects, whereas patients were treated as random effects to account for inter‐individual variability. The residual covariance structure was modeled using compound symmetry. All statistical analyses were conducted using R software (version 4.3.1, R Foundation for Statistical Computing). The LMM was fitted using the lme4 package, and statistical significance was set at a two‐sided *p* value < 0.05. The same statistical approach was applied to analyze the HAMD‐17 subdimensions across the two groups using LMMs. LMMs were selected to address the repeated measures nature of our data, as they can account for intra‐subject correlations and inter‐group variability, while also handling missing data. To account for multiple comparisons, statistical significance for these analyses was defined as a two‐sided *p* value < 0.0125, corresponding to the Bonferroni correction applied to the tests performed. This adjustment ensures the robustness of the results and accounts for the increased risk of Type I errors due to multiple comparisons.

#### 2.4.3 Survival Analysis

Kaplan–Meier survival curves were generated to compare the time to full remission (defined as an HAMD‐17 score below 7) between the two groups (Cox 1972). Log‐rank tests were conducted to statistically compare the survival curves. To account for potential baseline differences between the groups, a Cox proportional hazards regression model was employed to analyze remission time while adjusting for potential confounders. The Cox regression model initially included multiple candidate variables, and stepwise regression was applied to identify significant predictors for the final model. All survival analyses were conducted using R software (version 4.3.1, R Foundation for Statistical Computing). Kaplan–Meier analysis was performed using the survival and survminer packages, whereas the Cox proportional hazards regression model was fitted using the survival package. Survival analysis was employed to evaluate the time‐dependent nature of treatment responses, which is particularly suitable for understanding the effects of ECT over time. Statistical significance was defined as a two‐sided *p* value < 0.05.

#### 2.4.4 Linear Regression

Separate linear regression analyses were performed for the younger and older groups to explore the relationship between baseline and first post‐ECT depression scores and the number of subsequent interventions needed. In this analysis, only patients who achieved remission were included, whereas those who did not were excluded. For the analysis of first post‐ECT depression scores and subsequent treatment requirements, patients who required only a single intervention were also excluded. All regression analyses were conducted using SPSS version 26.0. Statistical significance was defined as a two‐sided *p* value < 0.05.

## 3 Results

### 3.1 Baseline Characteristics

A total of 132 patients experiencing depressive episodes were divided into two age‐based groups: younger (<30 years, *n* = 69) and older (≥30 years, *n* = 63). At baseline, both groups had comparable HAMD‐17 scores (mean ± standard deviation [SD]: 24.74 ± 5.77 vs. 24.27 ± 5.46, *p* > 0.05). Significant differences were observed in demographic and clinical characteristics, including age (mean ± SD: 20.98 ± 3.84 vs. 46.00 ± 5.25 years, *p* < 0.001), the presence of self‐harm behaviors (36.2% vs. 3.2%, *χ* ^2^ = 22.118, *p* < 0.001), suicidal attempts (46.4% vs. 28.6%, *χ* ^2^ = 4.437, *p* = 0.035), years of education (mean ± SD: 12.0 ± 2.0 vs. 8.0 ± 1.5 years, *U* = 3266, *p* < 0.001), and duration of illness (median [interquartile range, IQR]: 36 [12–60] vs. 84 [36–156] months, *U* = 1226, *p* < 0.001). As expected, the mean age in the older group was 25 years higher than in the younger group, reflecting the age‐based group allocation of the study. Similarly, the longer illness duration in the older group is consistent with their old age. Furthermore, significant differences were observed in the use of antidepressant medications between groups. The use was significantly different in the case of selective serotonin reuptake inhibitors (SSRIs) (29 vs. 15, *χ* ^2^ = 4.919, *p* = 0.027), serotonin–norepinephrine reuptake inhibitors (SNRIs) (29 vs. 39, *χ* ^2^ = 5.209, *p* = 0.022), and norepinephrine and specific serotonergic antidepressants (NaSSAs): 4 vs. 12 (*p* = 0.031) (Table 1).

**TABLE 1:** Demographic and clinical characteristics of younger and older groups.

	Younger group	Older group	Test statistic	*p* value
Unipolar depression	60 (87.0)	57 (90.5)	*U* = 0.131^c^	0.717
Female	47 (68.1)	44 (69.8)	*χ* ^2^_1_ = 0.046^b^	0.831
Participants with self‐injurious	25 (36.2)	2 (0.03)	*χ* ^2^_1_ = 22.118^b^	<0.001
Participants with suicide attempts	32 (46.4)	18 (28.6)	*χ* ^2^_1_ = 4.437^b^	0.035
Participants with psychiatric symptoms^a^	18 (26.1)	12 (19.0)	*χ* ^2^_1_ = 0.929^b^	0.335
Participants with medical comorbidities	3 (0.04)	7 (0.11)	*χ* ^2^_1_ = 1.294^b^	0.255
Family history of mental illness	3 (0.04)	7 (0.11)	*χ* ^2^_1_ = 1.294^b^	0.255
Age (years), median (IQR)	21.0 (18.0, 24.0)	46.0 (35.5, 51.0)	*U* = 0^c^	<0.001
Years of education (years), median (IQR)	12.0 (10.5, 15.0)	8.0 (7.0, 11.5)	*U* = 3266^c^	<0.001
Duration of illness (months), median (IQR)	36.0 (12.0, 60.0)	84.0 (36.0, 156.0)	*U* = 1226^c^	<0.001
Duration of current episode (days), median (IQR)	90.0 (30.0, 210.0)	90.0 (30.0, 180.0)	*U* = 2172^c^	0.996
Baseline HAMD‐17 score, mean (SD)/median (IQR)	24.74 (5.77)	24.27 (5.46)	*t*_129.01_ = 0.479^a^	0.633
Medicine category				
SSRIs (yes/no)	29	15	*χ* ^2^_1_ = 4.919^b^	0.027
SNRIs (yes/no)	29	39	*χ* ^2^_1_ = 5.209^b^	0.022
SARIs (yes/no)	3	3	—^d^	1
NaSSAs (yes/no)	4	12	—^d^	0.031
NDRIs (yes/no)	0	0	—	NA
TCAs (yes/no)	0	0	—	NA
Antipsychotics (yes/no)	59	58	*χ* ^2^_1_ = 1.405^b^	0.236
Anticonvulsant (yes/no)	34	21	*χ* ^2^_1_ = 3.443^b^	0.064
Anti‐anxiety (yes/no)	5	11	*χ* ^2^_1_ = 3.225^b^	0.073
Lithium carbonate (yes/no)	0	0	—	NA
Non‐benzodiazepine hypnotic (yes/no)	26	29	*χ* ^2^_1_ = 0.945^b^	0.331

Abbreviations: HAMD‐17, 17‐item Hamilton Depression Rating Scale; NA, not applicable; NaSSAs, norepinephrine and specificity serotonergic antidepressants; NDRIs, norepinephrine‐dopamine reuptake inhibitors; SARIs, serotonin antagonist/reuptake inhibitors; SD, standard deviation; SNRIs, serotonin–norepinephrine reuptake inhibitors; SSRIs, selective serotonin reuptake inhibitors; TCAs, tricyclic antidepressants.

^a^ Two‐sample *t*‐test without assuming equal variances.

^b^ Pearson's chi‐squared test.

^c^ Mann–Whitney *U*‐tests.

^d^Fisher's exact test.

### 3.2 LMM Analysis

Longitudinal data from all 132 patients (1056 follow‐up data points) were analyzed using LMM. An autoregressive correlation structure was applied to account for repeated measures within subjects, and the model was selected on the basis of the Akaike information criterion. The LMM revealed that each ECT session was associated with an average reduction of 2.166 points in total HAMD‐17 scores (*β* = −2.166, *z* = −28.503, *p* < 0.001). Additionally, a significant interaction between age group and ECT intervention was observed (*β* = −0.463, *z* = 4.408, *p* < 0.001), indicating that ECT was more effective in reducing depression scores in older adults compared to younger individuals. Patients with self‐harm behaviors exhibited significantly higher depression scores (*β* = 3.208, *z* = 2.424, *p* = 0.015). No other variables demonstrated statistically significant effects (Table 2).

**TABLE 2:** Linear mixed‐effects model (LMM) analysis of electroconvulsive therapy (ECT)’s impact on depression scores: assessing group differences and the role of baseline variables with notable disparities.

	Coef.	Std. err.	*z* value	*p* value	[0.025, 0.975]	
Intercept	21.410	2.024	10.578	0	17.443	25.377
Group	−2.003	1.325	−1.511	0.131	−4.600	0.595
Session	−2.166	0.076	−28.503	0	−2.315	−2.017
Group–Session	0.463	0.105	4.408	0	0.257	0.669
Duration of illness	−0.005	0.007	−0.740	0.460	−0.020	0.009
Years of education	0.010	0.144	0.071	0.943	−0.273	0.293
Self‐injurious (yes/no)	3.208	1.324	2.424	0.015	0.614	5.802
Suicide attempts (yes/no)	−0.787	1.005	−0.783	0.434	−2.757	1.183
SSRIs	0.726	1.195	0.607	0.544	−1.617	3.069
SNRIs	−1.203	1.109	−1.085	0.278	−3.376	0.970
NaSSAs	−0.377	1.541	−0.245	0.807	−3.397	2.644

*Note*: The table presents the results of the LMM analysis evaluating the impact of ECT on depression scores. The coefficients (coef.) indicate the effect sizes for each predictor variable, with standard errors (std. err.), *z* values, and *p* values reported for significance testing. A significant interaction between session and group (*p* < 0.001) suggests that the effect of ECT on depression scores differs by group. Notably, self‐injurious behavior is associated with a significant increase in depression scores (*p* = 0.015). The intercept represents the predicted depression score when all predictors are set to zero. Confidence intervals (0.025, 0.975) for each coefficient are also included to assess the precision of the estimates.

Abbreviations: NaSSAs, norepinephrine and specific serotonergic antidepressants; SNRI, serotonin–norepinephrine reuptake inhibitor; SSRIs, selective serotonin reuptake inhibitors.

Analysis of the four symptom dimensions using LMM further revealed that the number of ECT sessions was significantly associated with reductions in scores across all dimensions (Table 3):

**TABLE 3:** Linear mixed‐effects model (LMM) model results across four dimensions for the effects of electroconvulsive therapy (ECT) interventions in two groups.

Dimension	Variable	Coef.	Std. err.	*z* value	*p* value	[0.025, 0.975]	
1	Intercept	8.624	1.678	5.140	0.000	5.336	11.913
1	Session	0.635	0.693	0.916	0.360	−0.723	1.993
1	Group	−0.741	0.089	−8.346	0.000	−0.915	−0.567
1	Session–Group	−0.100	0.057	−1.760	0.078	−0.212	0.011
2	Intercept	4.316	1.175	3.674	0.000	2.014	6.619
2	Session	0.975	0.484	2.017	0.044	0.027	1.923
2	Group	−0.137	0.059	−2.321	0.020	−0.253	−0.021
2	Session–Group	−0.250	0.038	−6.582	0.000	−0.324	−0.175
3	Intercept	2.870	0.719	3.993	0.000	1.461	4.279
3	Session	0.030	0.299	0.100	0.920	−0.557	0.617
3	Group	−0.213	0.043	−4.975	0.000	−0.296	−0.129
3	Session–Group	−0.053	0.027	−1.948	0.051	−0.107	0.000
4	Intercept	1.631	0.591	2.761	0.006	0.473	2.788
4	Session	0.354	0.243	1.453	0.146	−0.123	0.831
4	Group	−0.153	0.030	−5.047	0.000	−0.212	−0.094
4	Session–Group	−0.058	0.019	−2.995	0.003	−0.096	−0.020

*Note*: This table presents key results from four LMM models that examine the effects of ECT interventions on four symptom dimensions in two patient groups. Each model includes covariates such as self‐injury and suicide to assess their impact on the outcome variable. Because the full table is lengthy, only the core findings are shown here. The coefficients (coef.) represent effect sizes for each variable, with standard errors (std. err.), *z* values, and *p* values provided to indicate the significance of these effects. Notably, significant interactions between session and group are found in dimensions 2 (*p* < 0.001) and 4 (*p* = 0.003), suggesting differential impacts of ECT based on group membership (Bonferroni correction was adopted, *p* < 0.0125 was statistically significant). Confidence intervals ([0.025, 0.975]) are included to indicate the precision of each estimate.

Dimension 1 (core depressive symptoms): Each additional ECT session was associated with a reduction of 0.741 points (*z* = −8.346, *p* < 0.001). The interaction between age group and ECT sessions was not significant (*β* = −0.100, *p* = 0.078).

Dimension 2 (anxiety): Each ECT session reduced anxiety scores by 0.137 points (*z* = −2.321, *p* = 0.020), with a significant interaction between age group and ECT sessions (*β* = −0.250, *p* < 0.001), indicating greater anxiety reduction in the older group.

Dimension 3 (insomnia): An increase in ECT sessions was associated with a reduction of 0.213 points in insomnia scores (*z* = −4.975, *p* < 0.001). The interaction between age group and ECT sessions was not significant (*β* = −0.053, *p* = 0.051).

Dimension 4 (somatic symptoms): Each ECT session reduced somatic symptom scores by 0.153 points (*z* = −5.047, *p* < 0.001), with a significant interaction effect (*β* = −0.058, *p* = 0.003), suggesting differential somatic symptom improvement between age groups.

These findings indicate that ECT effectively reduces depressive symptoms across all dimensions. However, the extent of reduction in anxiety and somatic symptoms varies significantly between age groups, with older adults showing greater improvements in these domains.

**FIGURE 1:**
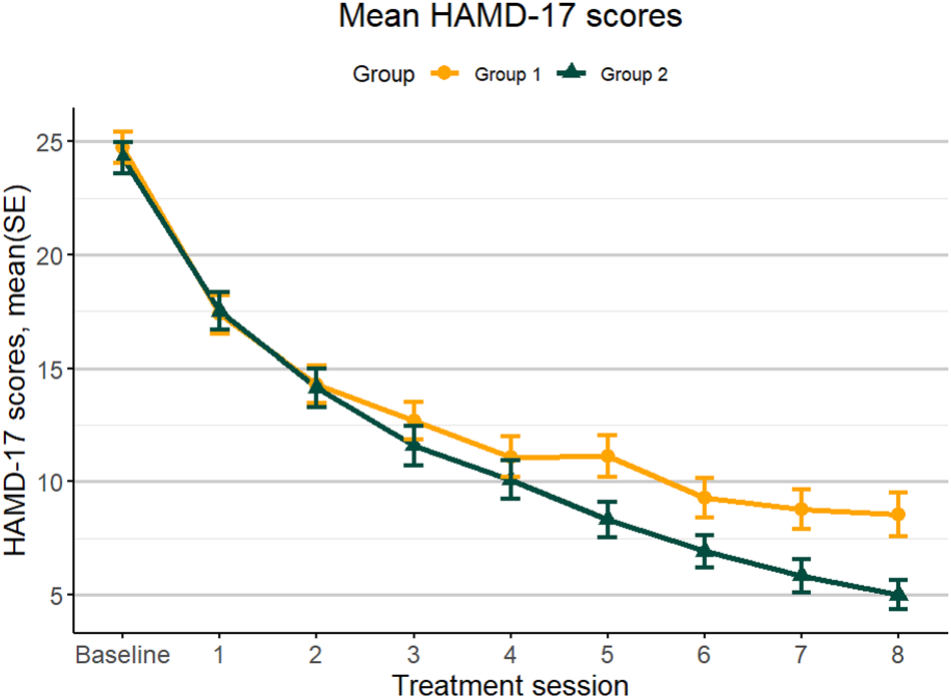
Trajectories of average HAMD‐17 total scores in the younger group (Group 1) and the older group (Group 2) across treatment sessions. HAMD‐17, 17‐item Hamilton Depression Rating Scale; SE, standard errors.

**FIGURE 2:**
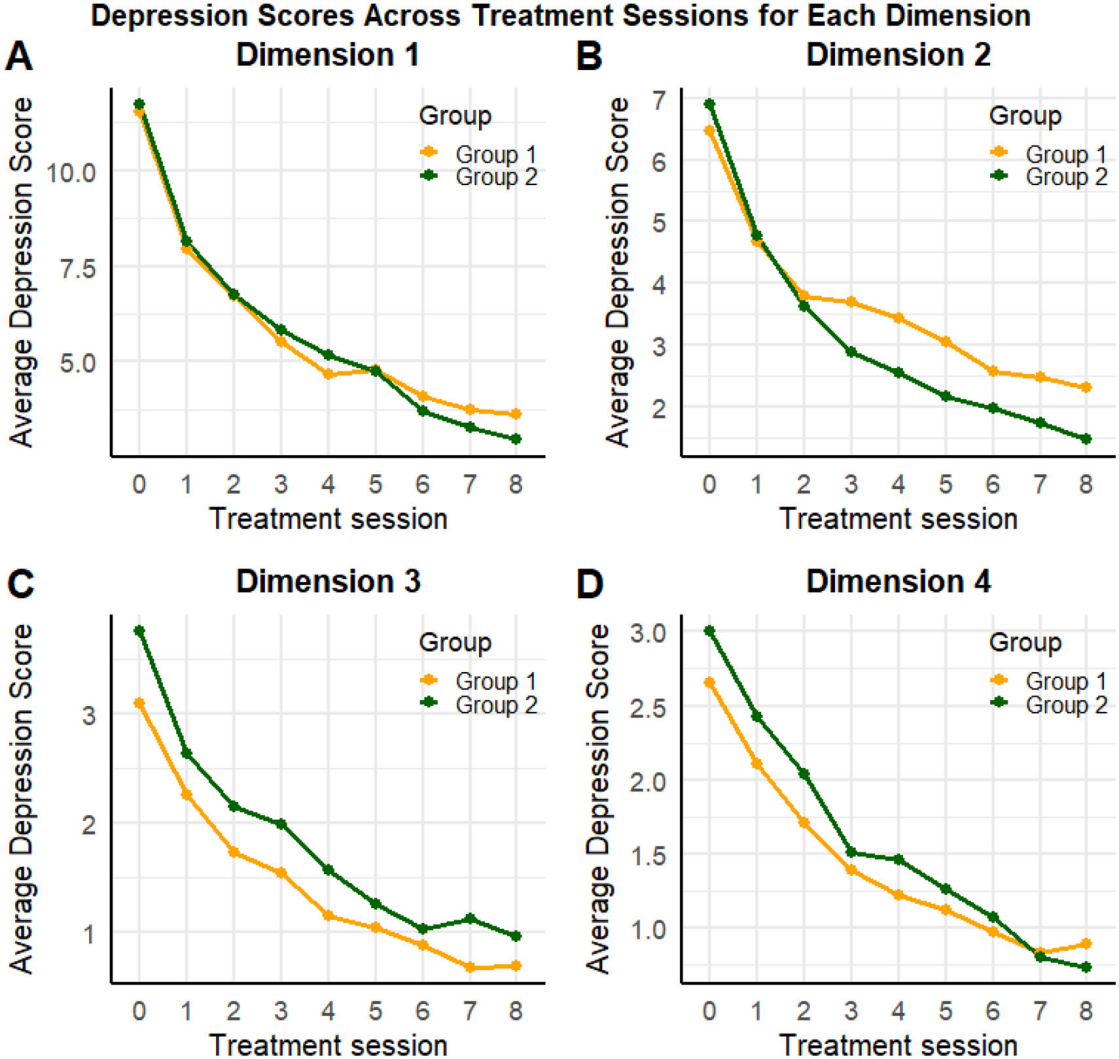
The trajectory of depression scores in the younger group (Group 1) and the older group (Group 2) across four dimensions over the course of treatment sessions. (A) Dimension 1: Core depressive symptoms (HAMD items 1, 2, 3, 7, 8)； (B) Dimension 2: Anxiety symptoms (HAMD items 9, 10, 11, 15, 17)； (C) Dimension 3: Insomnia symptoms (HAMD items 4, 5, 6) (D)； Dimension 4: Somatic symptoms (HAMD items 12, 13, 14, 16).

To present the data more clearly, Figure 1 displays the trajectories of mean HAMD‐17 scores and their standard errors (SE) for both groups of patients following each ECT session. Figure 2 illustrates the trajectories of mean values and SE for the four dimensions of the HAMD‐17 in both groups after ECT sessions.

### 3.3 Survival Analysis

**FIGURE 3:**
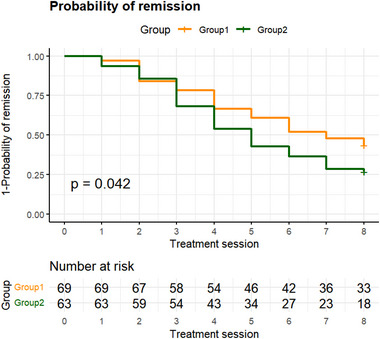
The Kaplan–Meier curve illustrates the probability of remission as a function of treatment time. The older group (Group 2) experienced a significantly faster time to remission compared to the younger group (Group 1) (*χ* ^2^ = 4.1, *p* = 0.042).

Kaplan–Meier survival curves illustrated the probability of remission over the course of ECT treatment (Figure 3). The median number of ECT sessions required to achieve remission was seven for the younger group and five for the older group. The log‐rank test confirmed that the older group achieved full remission significantly faster than the younger group (*χ* ^2^ = 4.100, *p* = 0.042), suggesting a more rapid therapeutic response to ECT in older adults.

**FIGURE 4:**
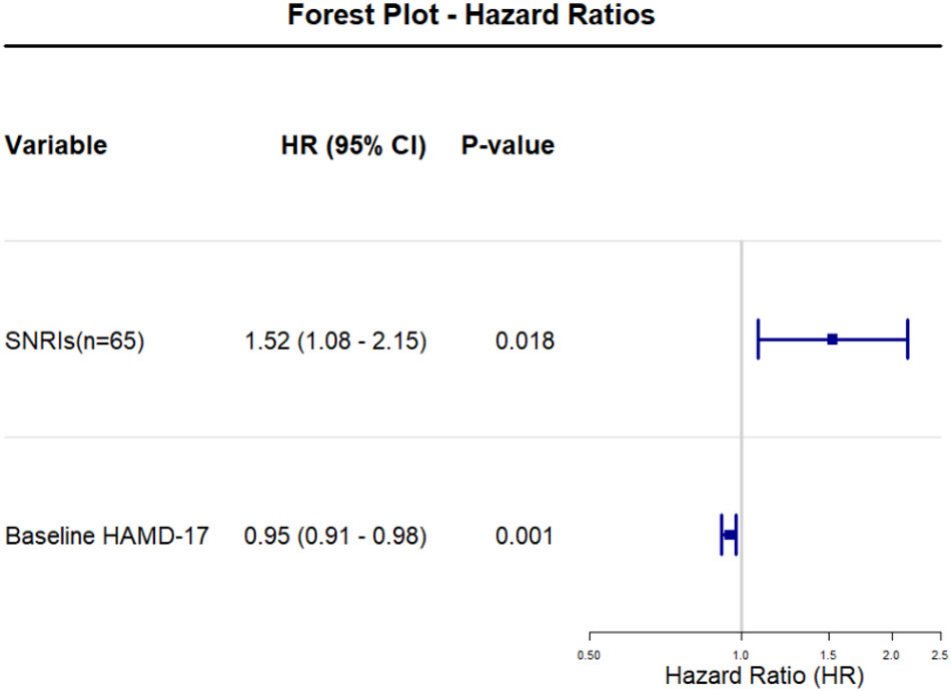
Factors associated with remission: hazard ratios from the Cox proportional hazards model. HAMD‐17, 17‐item Hamilton Depression Rating Scale; SNRIs, serotonin–norepinephrine reuptake inhibitors.

To further explore factors influencing remission, Cox proportional hazards regression analysis was performed. Initial models included multiple candidate predictors, and stepwise selection was used to refine the model. The final model identified two significant predictors (Figure 4): Baseline HAMD‐17 score: Higher baseline scores were associated with a lower likelihood of remission (hazards ratio [HR] = 0.945, 95% confidence interval [CI]: 0.914–0.977, *p* = 0.001), indicating that greater symptom severity at baseline predicted a longer time to remission; and SNRIs use: Patients using SNRIs showed a significantly higher likelihood of remission (HR = 1.52, 95% CI: 1.075–2.151, *p* = 0.018).

### 3.4 Linear Regression Analysis

In the older group (≥30 years), baseline HAMD‐17 total scores significantly predicted the number of subsequent ECT treatments required (*β* = 0.114, *R* ^2^ = 0.125, *p* = 0.016). Higher baseline HAMD‐17 scores predicted a greater number of ECT sessions required for remission. In contrast, in the younger group (<30 years), baseline scores did not significantly predict subsequent treatments (*β* = 0.072, *R* ^2^ = 0.037, *p* = 0.239).

**FIGURE 5:**
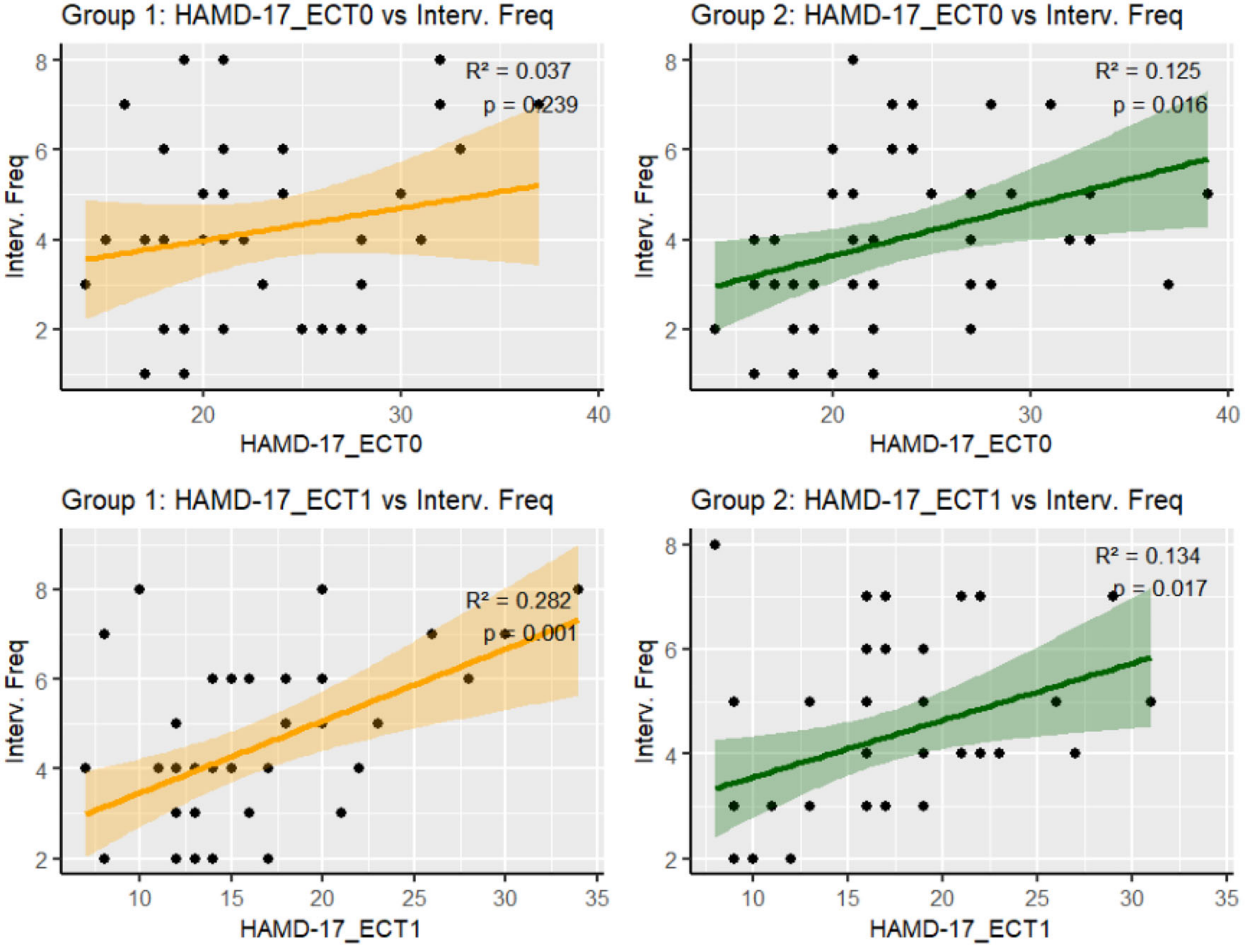
Relationship between HAMD‐17 and interv. freq. HAMD‐17, 17‐item Hamilton Depression Rating Scale. Interv. freq., subsequent intervention frequency. ECT, electroconvulsive therapy; HAMD‐17, 17‐item Hamilton Depression Rating Scale.

After the first ECT intervention, both groups showed significant predictive values for subsequent treatments. Specifically, in the younger group, the first post‐ECT HAMD‐17 score was a strong predictor (*β* = 0.160, *R* ^2^ = 0.282, *p* = 0.001), whereas in the older group, the first post‐ECT score also remained significant (*β* = 0.109, *R* ^2^ = 0.134, *p* = 0.017). These results indicate that the predictive value of depression scores for subsequent treatment requirements is more robust following the first post‐ECT session and differs across age groups (Table 4 and Figure 5).

**TABLE 4:** Linear regression of baseline and post‐1 electroconvulsive therapy (ECT) depression scores on subsequent interventions.

Group	Time point	*R* ^2^	Coef.	Std. err.	*t* value	*p* value	[0.025, 0.975]	
1	Baseline	0.037	0.072	0.06	1.20	0.239	−0.050	0.194
1	ECT1	0.282	0.160	0.043	3.71	0.001	0.073	0.248
2	Baseline	0.125	0.114	0.045	2.51	0.016	0.022	0.205
2	ECT1	0.134	0.109	0.044	2.49	0.017	0.021	0.197

*Note*: This table presents the results of linear regression analyses evaluating the relationship between baseline and post‐first ECT depression scores and subsequent intervention counts for two patient groups. The *R* ^2^ values indicate the proportion of variance explained by the model. In Group 1, the baseline score had a low predictive ability (*R* ^2^ = 0.037), whereas the post‐ECT score significantly predicted subsequent interventions (*R* ^2^ = 0.282, *p* = 0.001). For Group 2, the baseline score demonstrated a moderate predictive ability (*R* ^2^ = 0.125, *p* = 0.016), and the post‐ECT score also showed significant predictive capability (*R* ^2^ = 0.134, *p* = 0.017). Confidence intervals ([0.025, 0.975]) are provided for each coefficient to assess the precision of the estimates.

## 4 Discussion

This longitudinal study investigated the age‐dependent efficacy of ECT in patients with depression, focusing on symptom trajectories and treatment predictors. The results showed that each ECT session significantly alleviates depressive symptoms, with older patients experiencing a greater reduction in symptoms compared to younger patients in anxiety and somatic symptoms improvement. Older patients also demonstrate significantly faster rate of remission. However, multivariate Cox regression analysis revealed that baseline HAMD‐17 scores and the use of SNRIs were the primary predictors of remission, with age differences no longer being statistically significant. The study further indicated that age affects the ability of depression scores to predict the frequency of subsequent treatments. Notably, the depression score immediately following the first ECT session was a better predictor of subsequent treatment frequency than the baseline score, emphasizing the importance of early treatment response. This study provides the first systematic analysis of symptom trajectories and treatment predictors of ECT in patients with depression across different age groups. It particularly highlights the rapid response and remission patterns in older patients, filling a gap in the existing literature regarding age‐dependent efficacy trajectories and predictors while offering valuable insights for the clinical optimization of ECT protocols.

This study supports Hypothesis 1, confirming that age significantly influences the efficacy of ECT. Specifically, older patients exhibit a more pronounced reduction in HAMD‐17 scores. These findings are consistent with previous research (Heijnen et al. 2019; van Diermen et al. 2020). The impact of age on ECT efficacy may stem from neurobiological changes in older patients, including alterations in neurotransmitter levels and brain structure, which potentially enhance their responsiveness to ECT (Ousdal et al. 2022). Prior studies have demonstrated that the hippocampus and prefrontal cortex regions in older patients may undergo more significant neuroplastic changes during ECT, thereby improving treatment outcomes (Saberi et al. 2022; Yrondi et al. 2018).

The severity of initial depressive symptoms and the type of medication used are critical factors associated with the speed of remission. A lower baseline HAMD‐17 score may indicate milder depressive symptoms, making it easier for patients to achieve remission. Additionally, the use of SNRIs is positively correlated with remission, suggesting that these medications may offer superior efficacy in treating depression and warrant further clinical investigation. These results align with the findings of Nilsen et al. (2013) and Chen et al. (2007), who identified baseline symptom severity as a key predictor of treatment response. However, this study also highlights that relying solely on age as a prognostic indicator may be insufficient for comprehensively assessing treatment outlook of a patient. It is essential to integrate other clinical indicators for a more holistic evaluation (Loef et al. 2024). The observed disparities in SSRI/SNRI prescribing patterns in baseline data comparisons may reflect age‐specific clinical considerations. For older patients, clinicians may favor SNRIs due to their dual norepinephrine–serotonin reuptake inhibition, which could address comorbid somatic symptoms (e.g., chronic pain, fatigue) more effectively—a pattern common in aging populations (Rej et al. 2014). Our study also observed a greater comorbidity burden in the older cohort, without achieving statistical significance (Table 1).

In examining Hypothesis 2, we found that older patients experienced more significant improvements in both anxiety and somatic symptom dimensions following ECT. This may be attributed to superior emotional regulation capabilities in older patients (Sun et al. 2022). Typically, older individuals possess more extensive life experiences and mature coping strategies, enabling them to better adapt to and benefit from ECT (Santarnecchi et al. 2018). Furthermore, Winecoff et al. (2011) observe that compared to younger adults, older adults show reduced cognitive reappraisal‐related activation in the lateral prefrontal cortex, particularly in the left inferior frontal gyrus (LIFG). This finding suggests that the LIFG plays a critical role in the emotional regulation of older individuals, and we speculate that ECT‐induced changes in this region may differ between younger and older groups. Nevertheless, these hypotheses require further investigation. Future research should employ neuroimaging techniques to explore functional and structural brain changes in different age groups to better understand potential mechanisms.

When comparing baseline data between younger and older groups, significant differences were observed in educational level, illness duration, and the presence of self‐harm behaviors, all of which could influence ECT outcomes (Barnett et al. 2023; Kirov et al. 2021; Saunders and Smith 2016). LMM analysis indicated that self‐harm behavior had significant impact on ECT efficacy, with patients exhibiting self‐harm behaviors showing higher overall depression scores. This aligns with the previous research indicating that non‐suicidal self‐injury is associated with poorer ECT outcomes (Rootes‐Murdy et al. 2019). These results suggest that future studies should fully account for these confounding factors to assess the independent impact of age on ECT efficacy more accurately. Demographic profiling revealed a notably younger median age in our cohort compared to Western ECT service recipients (Luccarelli et al. 2020; Read et al. 2018), suggesting that this disparity may originate distinct sociocultural paradigms and mental healthcare delivery specificities in China. We propose two interconnected explanatory factors: (1) heightened mental health stigmatization among older adults (Tong et al. 2011), which may reduce ECT acceptance rates, and (2) regionally underdeveloped geriatric psychiatric infrastructure that limits treatment accessibility (Xia et al. 2021). These systemic barriers emphasize the need for tailored public health strategies and equitable clinical resource distribution to bridge therapeutic disparities in elderly depression care.

Consistent with Hypothesis 3, age not only affects changes in depression scores but also influences the ability of these scores to predict subsequent treatment needs. Specifically, baseline depression scores effectively predict the number of future ECT sessions required for older patients, whereas this predictive capability is not observed in younger patients. This indicates that age plays a crucial role in the symptomatic presentation and treatment response of depression. Alexopoulos (2005) noted that older patients with depression often exhibit more persistent and severe symptoms, potentially related to physiological aging and comorbidities. Consequently, baseline depression scores more accurately reflect illness severity and predict treatment needs in older patients. In contrast, depressive symptoms in younger patients are more susceptible to environmental stressors and social factors, resulting in greater symptom variability. Hammen (2018) demonstrates that psychosocial factors such as life events and social support play a more significant role in younger depressed patients, which may diminish the predictive efficacy of baseline depression scores for their future treatment needs. This aligns with the findings from the previous section of this study, where reductions in anxiety and somatization symptoms were less pronounced in the younger group compared to the older group.

Therefore, for older patients with depression, baseline depression scores can serve as an effective tool for assessing subsequent treatment needs, aiding clinicians in formulating individualized treatment plans. For younger patients, it may be necessary to comprehensively consider other assessment indicators, such as life stress, social support, and psychological resilience, to accurately predict their treatment requirements. This is consistent with the biopsychosocial medical model proposed by Engel (1977), which emphasizes the importance of comprehensive evaluation in disease management.

Moreover, unlike most studies that use baseline scores as predictive indicators (Mazza et al. 2020; Tunvirachaisakul et al. 2018), our research found that depression scores after the first ECT treatment are more effective in predicting future treatment needs. Specifically, a lower HAMD‐17 score after the first treatment is associated with fewer subsequent interventions, suggesting that early response can provide a solid foundation for customizing future treatment strategies (Carstens et al. 2021). This finding has significant clinical implications, especially considering that ECT typically involves multiple sessions. Early prediction of treatment outcomes can optimize treatment plans, minimize unnecessary sessions, and reduce side effects and costs (Argyelan et al. 2021; Selva‐Sevilla et al. 2016).

The results of this study offer new insights into the application of ECT in treating depression, particularly in managing patients of different age groups. Clinicians can adjust treatment plans based on early responses to enhance efficacy and reduce unnecessary interventions. However, several limitations warrant consideration. First, although our study divided participants into younger and older groups based on the median age (30 years) to balance group sizes statistically, this approach may not reflect clinically meaningful age categories (e.g., 18 and under, 18–64, 65+). Additionally, the older group (30+ years) encompasses a wide neurobiological and physiological range, suggesting future studies should stratify patients into narrower age brackets to better capture age‐related differences in ECT response. Second, the relatively modest sample size and single‐center design may constrain the generalizability of our findings; consequently, future investigations should incorporate larger scale, multicenter cohorts to validate these observations. Third, although the HAMD‐17 score is recognized as a valid measure of depression severity, it may not adequately capture the complex neural and biological factors influencing ECT efficacy. To address this, future research should integrate neuroimaging findings and serum biomarkers to identify multimodal predictors of remission. Fourth, although half‐age dosing was used in this study, threshold titration (1.5–2.0× seizure threshold) could allow more personalized treatment, highlighting the need for future studies to explore EEG monitoring combined with dose adjustment for improved accuracy. Finally, although this regimen was selected on the basis of institutional protocols aimed at achieving rapid depression improvement, limited evidence supports its superiority over thrice‐weekly schedules, underscoring the necessity for further research comparing efficacy and cognitive outcomes across regimens.

## 5 Conclusion

This study aimed to examine the influence of age on the efficacy of ECT and the prediction of treatment requirements in patients with depression. The findings demonstrate that age significantly impacts ECT outcomes, with older adults exhibiting more robust and favorable responses. Additionally, depression scores following the initial ECT session effectively predict the total number of treatments required, supporting the development of personalized ECT protocols based on early treatment response. These results underscore the importance of individualized treatment approaches in clinical practice to enhance therapeutic efficacy and optimize resource allocation. Future research should aim to validate the effectiveness of these personalized strategies and explore additional factors that influence the treatment responses, with the goal of continuously improving the therapeutic experience and quality of life for individuals suffering from depression.

## Author Contributions

**Wanling Huang**: conceptualization, methodology, writing–original draft, data curation, formal analysis, visualization, software. **Yang Ji**: conceptualization, methodology, writing–original draft, investigation. **Nanxue Duan**: data curation, visualization, formal analysis. **Hao Zheng**: investigation. **Rui Qian**: investigation. **Kai Wang**: funding acquisition, project administration, resources, validation. **Jianhong Li**: supervision, writing–review and editing, conceptualization. **Yanghua Tian**: funding acquisition, project administration, resources, conceptualization, supervision, writing ‐ review and editing.

## Disclosure

This work has not been previously published and is not currently under consideration in any other journal.

## Ethics Statement

This study was conducted in accordance with the Declaration of Helsinki and was approved by the Ethics Committee of Anhui Medical University (Approval No: 84230093).

## Consent

Written informed consent was obtained from all participants prior to their inclusion in the study.

## Conflicts of Interest

The authors declare no conflicts of interest.

### Peer Review

The peer review history for this article is available at https://publons.com/publon/10.1002/brb3.70487

## Data Availability

Due to patient confidentiality and ethical considerations, the datasets generated and analyzed during the current study are not publicly available. De‐identified data may be available from the corresponding author upon reasonable request and with appropriate institutional approvals.
